# Bile acid metabolism and invasion-related genes as therapeutic monitoring biomarkers in non-small cell lung cancer

**DOI:** 10.1007/s12672-025-03925-x

**Published:** 2025-11-10

**Authors:** Guangteng Wu, Lin Zhu, Feng Xue, Yanyi Zhao

**Affiliations:** 1 Department of Medical Oncology, The First Affiliated Hospital of Guilin Medical University, Guilin, China; 2 Department of Radiotherapy, The First Affiliated Hospital of Guilin Medical University, Guilin, China

**Keywords:** Non-small cell lung cancer, Bile acid metabolism, Invasion, Differentially expressed genes, Therapeutic monitoring model

## Abstract

Non-small cell lung cancer (NSCLC) persists as a major contributor to global cancer morbidity and mortality. This study explores the complex interplay between bile acid metabolism (BAM), tumor invasion, and the immune microenvironment in NSCLC pathogenesis. While traditionally known for digestive functions, bile acids are crucial signaling molecules, and their metabolic pathways, coupled with invasion-related genes (IRGs), are increasingly implicated in tumorigenesis. Using the NSCLC dataset GSE225620, we identified 109 differentially expressed genes at the intersection of BAM and invasion (BAM&IRDEGs). Functional analysis revealed their significant enrichment in metabolic pathways, including fatty acid metabolism, which are critical for shaping the tumor microenvironment and fostering aggressive growth. Immune infiltration analysis indicated significant remodeling, particularly highlighting a strong correlation between activated CD8⁺ T cells and central memory CD4⁺ T cells. Key genes, including the glutamine transporter *SLC1A5* and the fatty acid translocase *CD36*, emerged as critical nodes. Our analysis suggests these genes contribute to an immunosuppressive microenvironment by fueling tumor metabolic reprogramming and inducing CD8⁺ T cell dysfunction through altered lipid and glutamine metabolism. Leveraging these key genes, we established a therapeutic monitoring model using LASSO regression and support vector machine (SVM) algorithms. The model demonstrated robust performance in predicting treatment efficacy, underscoring its potential for monitoring therapeutic response and guiding individualized treatment decisions. Our findings highlight the clinical relevance of the metabolic-immune axis in NSCLC and propose that key BAM&IRDEGs, particularly *SLC1A5* and *CD36*, are not only potential therapeutic targets but also promising candidates for inclusion in liquid biopsy panels. Such non-invasive tools could enable dynamic monitoring of disease progression and early detection of relapse, paving the way for more precise NSCLC management.

**Supplementary Information:**

The online version contains supplementary material available at 10.1007/s12672-025-03925-x.

## Introduction

Cancer constitutes a complex and heterogeneous group of diseases, and the conceptual understanding of it has profoundly evolved from a tissue-centric to a molecular and ecosystem-based perspective. This paradigm shift has consequently reshaped therapeutic strategies, fostering a transition from generalized treatments to personalized medicine tailored to the specific genetic and molecular aberrations of an individual’s tumor [1]. This conceptual evolution underscores that cancer is not a static entity but rather a dynamic process characterized by significant intratumor heterogeneity and clonal evolution, which pose considerable challenges to conventional therapeutic strategies.

Non-small cell lung cancer (NSCLC) remains a significant global health burden, imposing substantial strain on healthcare systems and adversely affecting patient survival rates worldwide [2]. Characterized by high rates of morbidity and mortality, this malignancy presents considerable socioeconomic challenges in addition to its clinical ramifications. The current standard of care encompasses surgical intervention, chemotherapeutic regimens, radiation therapy, and molecularly targeted agents [3, 4]. Despite significant advancements in these therapeutic modalities, persistent limitations, including heterogeneous treatment responses and the development of acquired resistance, highlight the urgent need for novel therapeutic paradigms [5, 6]. This clinical imperative motivates our investigation into novel pathobiological mechanisms with the potential to yield more effective therapeutic targets.

This study investigates the interplay between genes related to bile acid metabolism and invasion (BAM&IRGs) within the context of NSCLC. A growing body of evidence implicates these genes in tumorigenesis and cancer progression [7, 8]. Indeed, preceding studies have established that systemic bile acid metabolism is dysregulated in NSCLC, identifying significant alterations in patient serum bile acid profiles and linking this aberrant metabolism to tumor progression and adverse clinical outcomes [9, 10]. Bile acids, traditionally recognized for their function in lipid digestion, are now appreciated as key modulators of various cellular processes, including proliferation, apoptosis, and inflammation, all of which are implicated in carcinogenesis [11, 12]. Furthermore, the influence of bile acid metabolism extends to the modulation of the tumor immune microenvironment. For instance, a recent study in liver cancer demonstrated that the intratumoral accumulation of bile acids can directly suppress the function of anti-tumor T cells, thereby impeding an effective immune response and facilitating immune escape [13]. Recent research has further underscored the crucial role of bile acid metabolism in lung cancer pathogenesis. For example, the membrane bile acid receptor *TGR5* has been shown to drive NSCLC progression by activating the *JAK2/STAT3* signaling pathway, thereby promoting cell proliferation and migration; its overexpression is also associated with a poor prognosis in patients with NSCLC [14]. Moreover, metabolomic analyses have revealed distinct alterations in the bile acid profiles of NSCLC patients, such as increased primary bile acids (e.g., cholic acid, taurocholic acid) and decreased secondary bile acids (e.g., lithocholic acid), with subtype-specific patterns observed between adenocarcinoma and squamous cell carcinoma [9]. Concurrently, invasion-related genes are recognized as pivotal drivers of tumor metastasis, a primary determinant of poor prognosis in NSCLC [15]. Collectively, these findings provide a compelling rationale for investigating BAM&IRGs as potential novel biomarkers and therapeutic targets in NSCLC.

To elucidate the role of BAM&IRGs in NSCLC, this study utilized a comprehensive bioinformatics pipeline, encompassing data normalization, differential expression analysis, Gene Ontology (GO) and Kyoto Encyclopedia of Genes and Genomes (KEGG) pathway enrichment analyses, and the construction of predictive models. This computational methodology is adept at interrogating large-scale datasets, enabling the identification of clinically pertinent biomarkers and pathways within complex biological systems. In exploring the nexus between bile acid metabolism and tumor progression, the choice of sample source is of paramount importance. Whole blood samples offer distinct advantages for clinical applications due to their accessibility and suitability for dynamic monitoring. This minimally invasive approach facilitates the longitudinal observation of disease progression and therapeutic response. Critically, the metabolic profile of whole blood, including bile acids, is reflective of the overall systemic state and is intricately correlated with immune cell function and the tumor immune microenvironment [16, 17]. Therefore, a study based on whole blood analysis is well-positioned not only to unveil key features of metabolic reprogramming and immune modulation in NSCLC but also to possess high potential for clinical translation into future diagnostic and personalized treatment strategies. The primary objective of this research is to identify key genes integral to the pathogenesis and prognosis of NSCLC, with a particular focus on those involved in bile acid metabolism and invasion. Notably, this study represents the first systematic investigation into the molecular interplay between these two biological processes in NSCLC. Furthermore, we aim to construct and validate a robust therapeutic monitoring model based on these identified genes. Such a model holds the potential to inform early detection, risk stratification, and the development of personalized therapeutic strategies for patients with NSCLC.

## Materials and methods

### Data acquisition and gene set curation

The transcriptomic dataset GSE225620 [18], pertaining to NSCLC, was acquired from the Gene Expression Omnibus (GEO) database [19]. Data retrieval was performed using the R package GEOquery (version 2.70.0) [20]. This dataset, derived from *Homo sapiens*, comprises a featureCounts matrix comparing gene expression profiles from 17 pre-treatment and 21 post-treatment whole blood samples. Detailed clinical characteristics of the samples are summarized in Table 1.

A comprehensive list of genes related to bile acid metabolism (BAMRGs) was curated from two primary sources. First, a search of the GeneCards database [21] using the keyword “Bile Acid Metabolism” yielded 135 protein-coding genes. Second, a literature search in PubMed identified an additional 481 BAMRGs from a published study [22]. After merging and removing duplicates from both sources, a final list of 530 unique BAMRGs was compiled for subsequent analysis (Table S1).

Similarly, a list of invasion-related genes (IRGs) was assembled. This involved querying the GeneCards database [21] with the term “Invasion,” which retrieved 10,861 protein-coding genes, and supplementing this list with 97 IRGs identified from a prior publication [23]. The combined dataset resulted in a total of 10,869 unique IRGs (Table S2).

To identify genes at the nexus of both biological processes, the curated lists of BAMRGs and IRGs were intersected. This intersection yielded 408 genes, hereafter referred to as BAM&IRGs, which were utilized for all downstream analyses. A complete list of these genes is provided in Table S3.

### Identification of differentially expressed genes associated with bile acid metabolism and invasion in NSCLC

Samples from the GSE225620 dataset were stratified into Pre-treatment and Post-treatment groups. Differential expression analysis was subsequently performed between these groups using the limma R package (Version 3.58.1). Differentially expressed genes (DEGs) were identified based on the criteria of an absolute log2 fold change (|logFC|) > 0 and a p-value < 0.05. Genes were classified as upregulated (logFC > 0) or downregulated (logFC < 0). The results of this analysis were visualized as a volcano plot generated with the ggplot2 R package (Version 3.4.4).

To identify bile acid metabolism and invasion-related differentially expressed genes (BAM&IRDEGs) in NSCLC, we performed an intersection analysis between the DEGs derived from GSE225620 and a pre-compiled list of known bile acid metabolism and invasion-related genes (BAM&IRGs). The resulting overlap was depicted in a Venn diagram. Furthermore, a heatmap was generated using the pheatmap R package (Version 1.0.12) to illustrate the expression patterns of the top 20 most significant BAM&IRDEGs.

### Functional and pathway enrichment analysis

To elucidate the biological roles of the identified BAM&IRDEGs, we conducted functional and pathway enrichment analyses. Specifically, GO [24] and KEGG [25] pathway analyses were performed using the clusterProfiler R package (Version 4.10.0) [26], Enrichment results were considered statistically significant if the p-value was less than 0.05 and the false discovery rate (FDR or q-value) was also less than 0.05.

### Establishment of therapeutic monitoring model for NSCLC

To identify key genes predictive of therapeutic response in NSCLC, BAM&IRDEGs from the GSE225620 dataset were first subjected to a preliminary screening using univariate logistic regression analysis. Only genes with a p-value less than 0.05 were retained for further analysis.

Subsequently, two distinct machine learning algorithms were applied in parallel to this filtered gene set for robust feature selection. First, a Least Absolute Shrinkage and Selection Operator (LASSO) regression model was implemented using the glmnet R package (family = “binomial”, set.seed(500)) [27]. Ten-fold cross-validation was employed to determine the optimal regularization parameter (lambda), which guided the selection of a core set of predictive genes, hereafter termed ‘Model Genes’. These genes were then used to calculate a risk score for each patient. Second, a Support Vector Machine (SVM) algorithm [28] was utilized to identify another set of feature genes, selected based on the criteria of maximizing predictive accuracy while minimizing the classification error rate.

Finally, to derive a high-confidence gene signature, we identified the intersection of the gene sets selected by both the LASSO and SVM models. This final, overlapping set was designated as the ‘Key Genes (mRNA)’ and formed the basis for all subsequent downstream analyses, thereby ensuring their robustness as potential biomarkers.

### Validation of the predictive model for NSCLC therapeutic response

To provide a quantitative and user-friendly tool for clinical application, a nomogram was developed based on the identified Key Genes using the rms R package. This nomogram graphically represents the logistic regression model, allowing for the prediction of therapeutic response by assigning a point value to the expression level of each Key Gene and summing these points to obtain a total score.

The model’s calibration was assessed by plotting a calibration curve, which evaluates the concordance between the nomogram-predicted probabilities and the actual observed outcomes. An ideal model exhibits a calibration curve that aligns closely with the 45-degree diagonal line.

Furthermore, the clinical utility and net benefit of the predictive model were evaluated through Decision Curve Analysis (DCA). The analysis was conducted using the ggDCA R package to generate DCA plots, which illustrate the potential clinical impact of using the model for decision-making across a range of threshold probabilities.

Finally, the discriminative performance of the model was quantified using Receiver Operating Characteristic (ROC) curve analysis with the pROC R package (Version 1.18.5). The Area Under the Curve (AUC) was calculated for the GSE225620 dataset to measure the model’s ability to accurately distinguish between the Pre- and Post-treatment groups.

### Characterization and validation of the key gene signature

First, to confirm the differential expression patterns of the Key Genes, their expression levels between the Pre- and Post-treatment groups in the GSE225620 dataset were visualized. Subsequently, the individual discriminatory power of each Key Gene was assessed through ROC curve analysis using the pROC R package (Version 1.18.5). The AUC was calculated for each gene to quantify its ability to distinguish between the two treatment states.

Furthermore, to explore the interrelationships within the gene signature, a Spearman correlation analysis was performed on the expression levels of the Key Genes. The resulting correlation matrix was visualized as a heatmap generated with the pheatmap R package (Version 1.0.12).

### Functional coherence analysis

To investigate the functional coherence of the Key Gene signature, a Gene Ontology (GO) semantic similarity analysis was performed using the GOSemSim R package. This method quantifies the functional relatedness between genes based on their shared GO annotations. Similarity scores were calculated for each gene pair across the three GO domains: Biological Process (BP), Cellular Component (CC), and Molecular Function (MF). The results were then visualized using the ggplot2 package to illustrate the functional relationships among the Key Genes.

### Immune infiltration analysis

To characterize the tumor immune microenvironment (TIME) associated with therapeutic response, single-sample Gene Set Enrichment Analysis (ssGSEA) [29] was employed to estimate the infiltration levels of various immune cell subtypes (e.g., Activated CD8 + T cells, Activated dendritic cells, and Regulatory T cells). This analysis generated an immune infiltration score for each cell type in every sample from the GSE225620 dataset.

Subsequently, the infiltration scores of these immune cell populations were compared between the Pre- and Post-treatment groups, and the results were visualized. Spearman’s correlation analysis was then performed to assess both the interrelationships among different immune cell types and the association between Key Gene expression and immune cell abundance. The correlation matrices were visualized as heatmaps and bubble plots using the pheatmap and ggplot2 R packages, respectively.

### Statistical analysis

R software (version 4.2.2) served as the computational environment for all data processing and statistical analyses in this study. Unless otherwise stated, comparisons between two continuous variables were executed by using the independent Student’s T-Test for normally distributed variables, whereas the Mann-Whitney U test (Wilcoxon Rank Sum Test) was utilized for non-normally distributed variables. For comparisons involving three or more groups, the Kruskal-Wallis test was implemented. Correlations among different molecules were evaluated by means of Spearman correlation analysis. All statistical tests were two-sided unless particularly indicated, with statistical significance determined as p-value < 0.05.

## Results

**Fig. 1:**
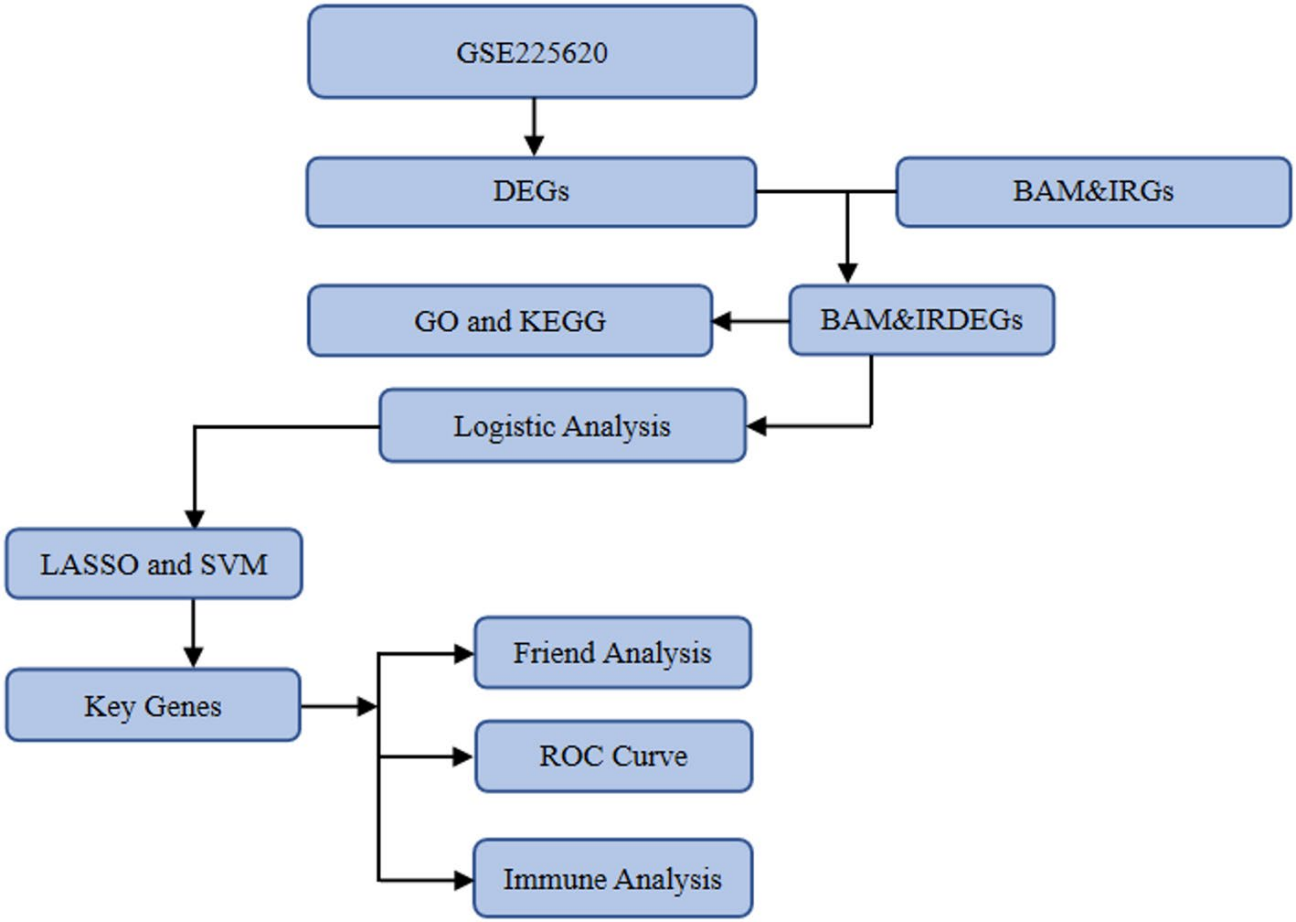
Technology Roadmap. *DEGs* Differentially Expressed Genes, *BAM&IRGs* Bile Acid Metabolism and Invasion-Related Genes, *BAM&IRDEGs* Bile Acid Metabolism and Invasion-Related Differentially Expressed Genes, *GO* Gene Ontology, *KEGG* Kyoto Encyclopedia of Genes and Genomes, *LASSO* Least Absolute Shrinkage and Selection Operator, *SVM* Support Vector Machine, *ROC* Receiver Operating Characteristic

### Data preprocessing and quality control

**Fig. 2:**
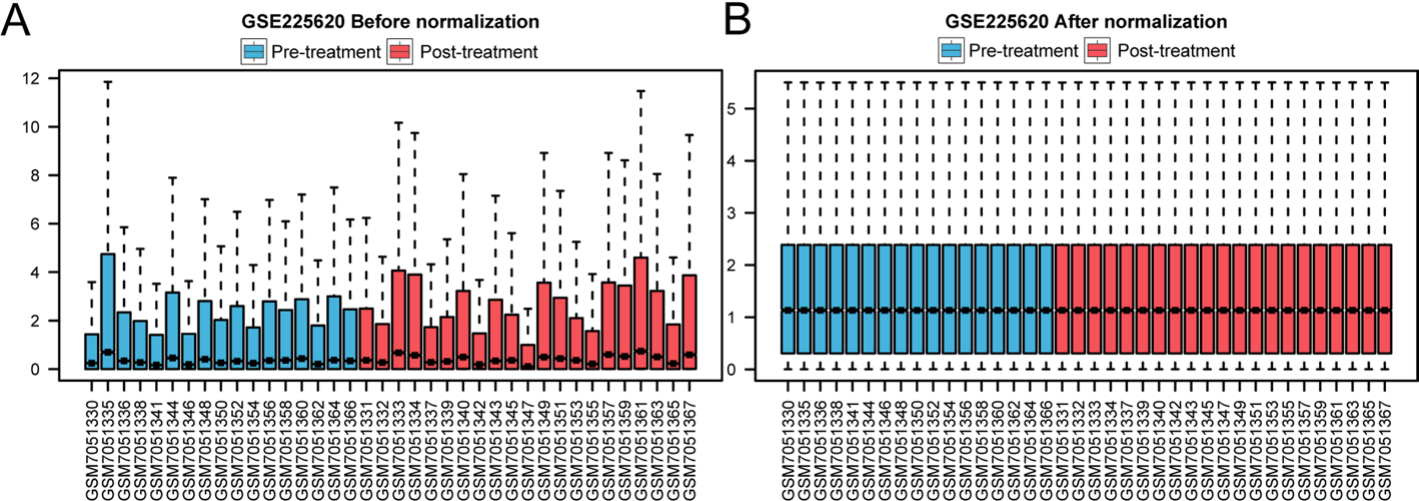
Dataset normalization before and after. **A** Box plot of the gene expression distribution in GSE225620 samples before normalization. **B** Box plot of the gene expression distribution in GSE225620 samples after normalization

The comprehensive analytical workflow for this bioinformatic study is depicted in Fig. 1. The GSE225620 dataset, stratified into Pre-treatment and Post-treatment cohorts, was subjected to a rigorous preprocessing pipeline. This included probe re-annotation and quantile normalization to ensure data quality and comparability across samples (Fig. 2A, B). Box plots generated before and after this process confirmed the effectiveness of the normalization, revealing a substantially more consistent expression distribution among samples post-processing.

### Identification of differentially expressed bile acid metabolism- and invasion-related genes (BAM&IRDEGs)

**Fig. 3:**
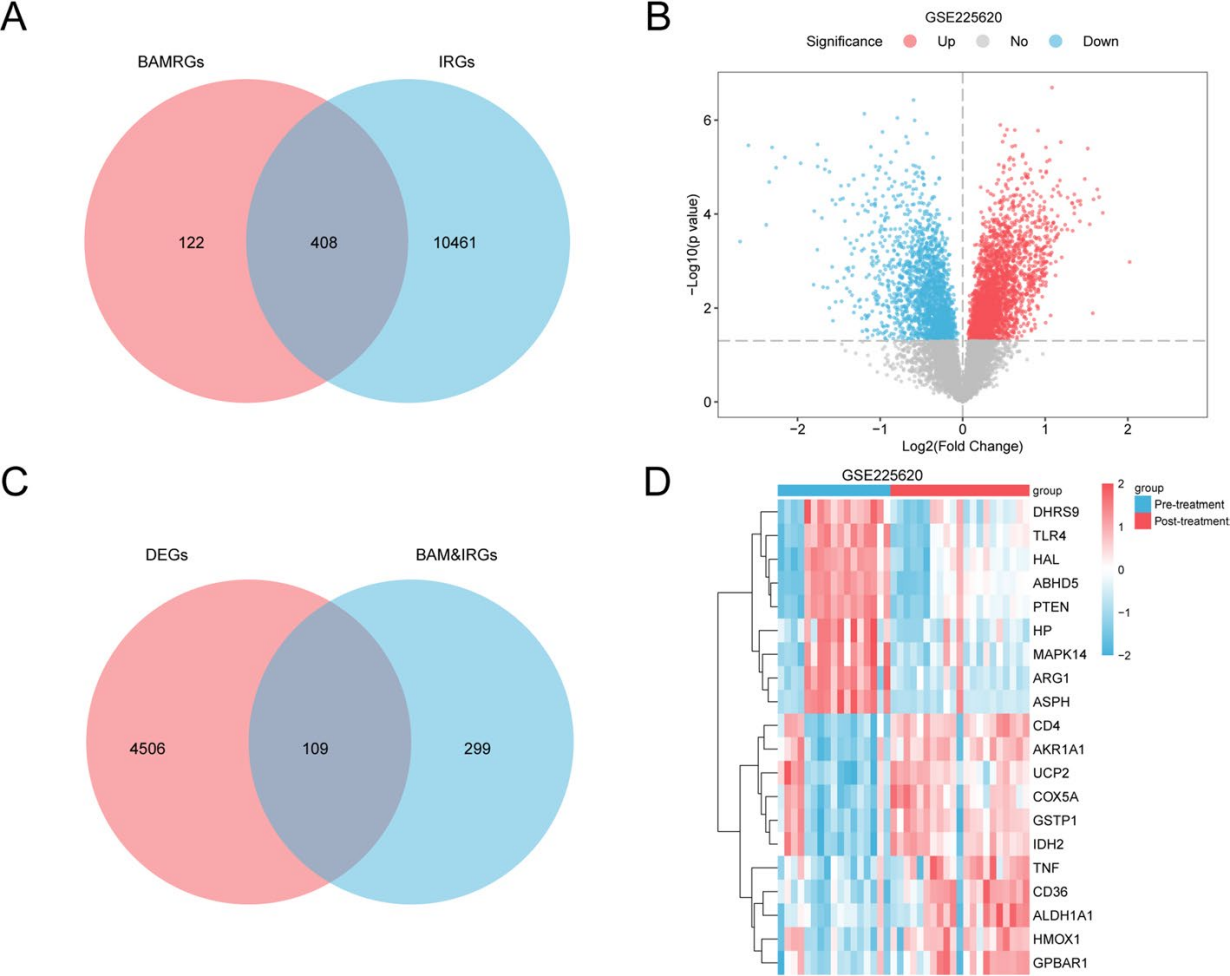
Identification of BAM&IRDEGs. **A** Venn diagram showing the intersection of BAMRGs and IRGs. **B** Volcano plot of DEGs in the GSE225620 dataset. **C** Venn diagram showing the intersection of DEGs and BAM&IRGs. **D** Heatmap of the top 20 differentially expressed BAM&IRDEGs. *BAMRGs* Bile Acid Metabolism-Related Genes, *IRGs* Invasion-Related Genes, *DEGs* Differentially Expressed Genes, *BAM&IRGs* Bile Acid metabolism-invasion-related Genes, *BAM&IRDEGs* Bile Acid Metabolism&Invasion-Related Differentially Expressed Genes

To identify genes at the nexus of bile acid metabolism and invasion, we first intersected the BAMRG and IRG sets. This analysis yielded 408 common genes, hereafter designated as BAM&IRGs (Fig. 3A).

Next, we performed a differential expression analysis on the GSE225620 dataset to compare the Post-treatment and Pre-treatment groups using the limma R package. A total of 4,615 differentially expressed genes (DEGs) were identified (criteria: |logFC| >0, *p* < 0.05), comprising 2,679 upregulated and 1,936 downregulated genes. A volcano plot was generated to visualize these expression changes (Fig. 3B).

By intersecting the 4,615 DEGs with the 408 previously identified BAM&IRGs, we pinpointed a core set of 109 BAM&IRDEGs (Fig. 3C; a complete list is available in Supplementary Table S4). This gene set included several key representatives, such as *FASN*,* CD36*,* ALAD*, and *SLC1A5*. The differential expression patterns of the top 20 BAM&IRDEGs between the two treatment groups were subsequently visualized via a heatmap (Fig. 3D).

### Functional enrichment analysis of BAM&IRDEGs

To elucidate the biological functions and signaling pathways associated with the 109 BAM&IRDEGs in NSCLC, we performed GO and KEGG pathway enrichment analyses. The detailed results are summarized in Table 2.

The GO analysis revealed that the BAM&IRDEGs were significantly enriched in specific terms across all three domains. Key BP included the fatty acid metabolic process, carboxylic acid catabolic process, organic acid catabolic process, small molecule catabolic process, and cellular lipid catabolic process. For CC, enrichments were predominantly found in the mitochondrial matrix, peroxisome, microbody, peroxisomal membrane, and microbody membrane. The primary MF identified were oxidoreductase activity (acting on the aldehyde or oxo group of donors with NAD or NADP as acceptor; acting on the CH-OH group of donors), monocarboxylic acid binding, and amide binding.

**Fig. 4:**
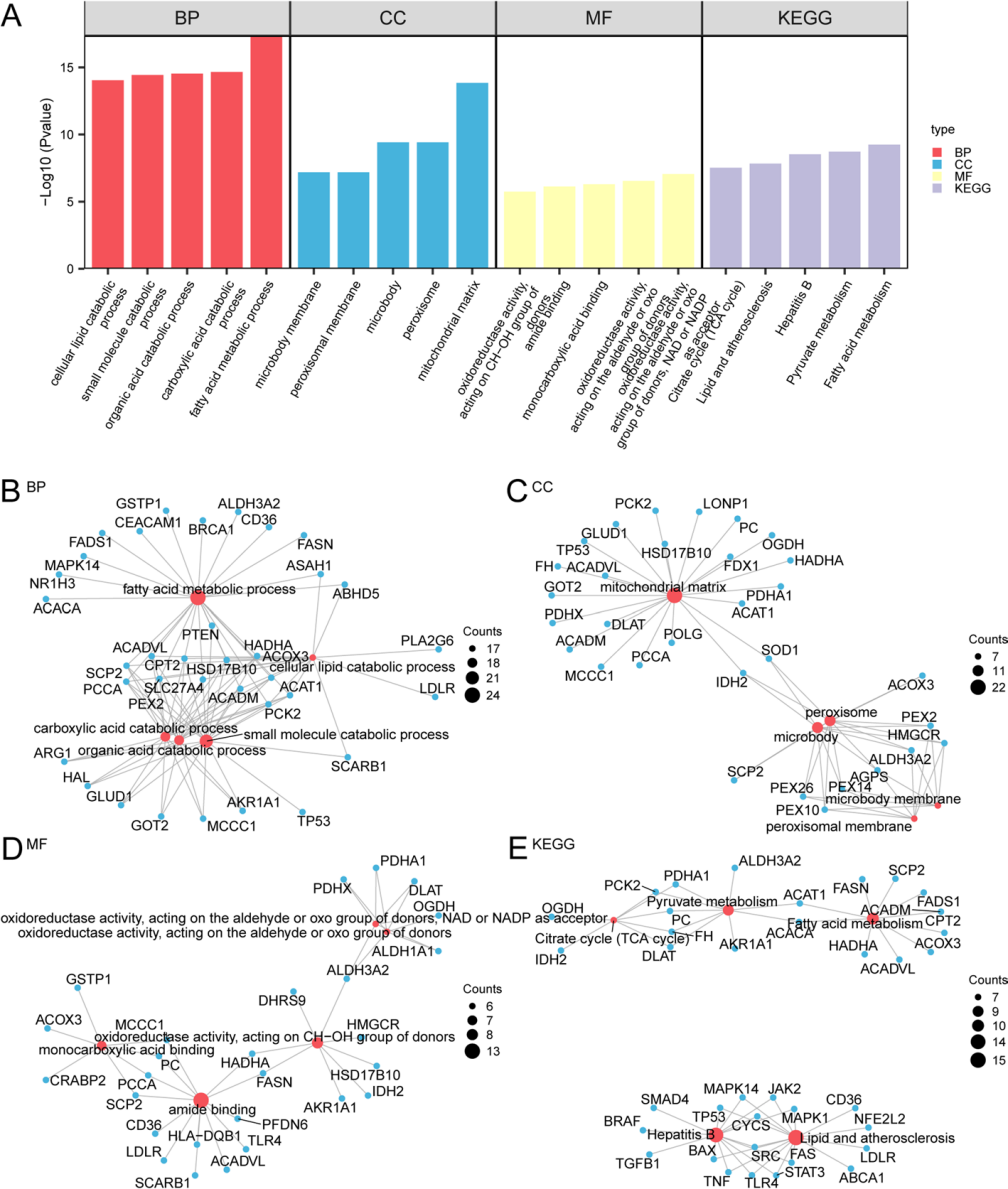
GO and KEGG enrichment analysis of BAM&IRDEGs. **A** Bar graphs showing the top enriched GO terms (BP, CC, MF) and KEGG pathways. The x-axis represents the enriched terms, and the y-axis shows the -Log10(P-value). **B**–**E**. Network diagrams illustrating the relationships between genes and enriched terms for BP (**B**), CC (**C**), MF (**D**), and KEGG (**E**). Red nodes represent items, blue nodes represent molecules, and the lines represent the relationship between items and molecules. *BAM&IRDEGs* Bile Acid metabolism-invasion-related Differentially Expressed Genes, *GO* Gene Ontology, *KEGG* Kyoto Encyclopedia of Genes and Genomes, *BP* Biological Process, *CC* Cellular Component, *MF* Molecular Function. The screening criteria for GO and KEGG enrichment analysis were p value < 0.05 and FDR value (q value) < 0.05

Similarly, KEGG pathway analysis indicated that these genes were enriched in pathways including Fatty acid metabolism, Pyruvate metabolism, Hepatitis B, Lipid and atherosclerosis, and the Citrate cycle (TCA cycle). The results from both the GO and KEGG enrichment analyses are presented as bar graphs for clear visualization (Fig. 4A).

Furthermore, we constructed network diagrams based on the GO and KEGG analyses to illustrate the intricate relationships between the genes and their corresponding enriched terms for BP, CC, MF, and KEGG pathways (Fig. 4B–E). In these networks, the connecting lines represent the association between a gene and an annotated term, while the node size is proportional to the number of genes involved in that term.

### Construction of therapeutic monitoring model for NSCLC

To identify candidate biomarkers for a therapeutic monitoring model in NSCLC, we initially screened the 109 BAM&IRDEGs using univariate logistic regression analysis. This preliminary screening identified 101 genes as being statistically significant (*P* < 0.05), the details of which are provided in Supplementary Table S4.

**Fig. 5:**
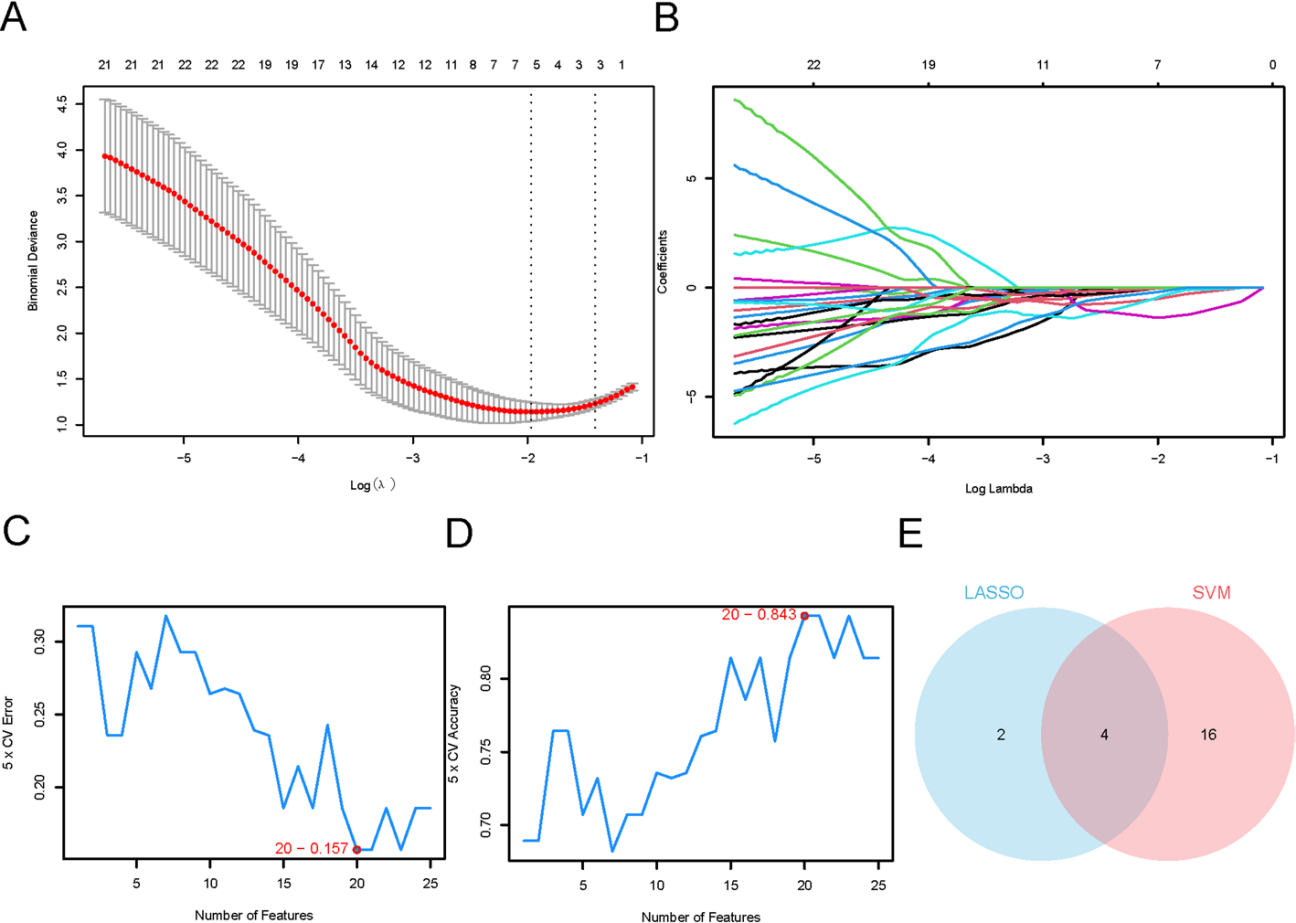
Construction of a therapeutic monitoring model for NSCLC. **A** LASSO regression model plot of BAM&IRDEGs in dataset GSE225620. **B** Variable trajectory plot of LASSO therapeutic monitoring model. **C** The number of genes with the lowest error rate obtained by the SVM algorithm. **D** The number of genes with the highest accuracy obtained by the SVM algorithm. **E** Venn diagram of intersection between LASSO algorithm and SVM algorithm. *LASSO* Least Absolute Shrinkage and Selection Operator, *SVM* Support Vector Machine, *BAM&IRDEGs* Bile Acid Metabolism&Invasion-Related Differentially Expressed Genes

To further refine this candidate pool, we employed two distinct machine learning approaches. First, a LASSO regression model was constructed, which pinpointed six key BAM&IRDEGs: *CD36*,* ALAD*,* CD4*,* FASN*,* ACP5*, and *SLC1A5* (Fig. 5A, B). Second, we developed a SVM model. An analysis of its error and accuracy rates revealed that the SVM model achieved optimal performance in distinguishing between treatment groups when using a specific set of 20 genes: *ALAD*,* HGF*,* SLC7A1*,* SLC1A5*,* CPT2*,* FASN*,* SLC3A2*,* PC*,* ACOX3*,* YY1*,* CD36*,* CPOX*,* ASPH*,* PTEN*,* FADS1*,* COX5A*,* PDHX*,* ALDH1A1*,* MAPK1*, and *SOD1* (Fig. 5C, D).

To isolate the most robust and consistently performing biomarkers, we determined the intersection of the gene sets derived from both the LASSO and SVM models (Fig. 5E). This approach yielded four key genes for subsequent investigation: *CD36*,* ALAD*,* FASN*, and *SLC1A5.*

### Validation of therapeutic monitoring model for NSCLC

**Fig. 6:**
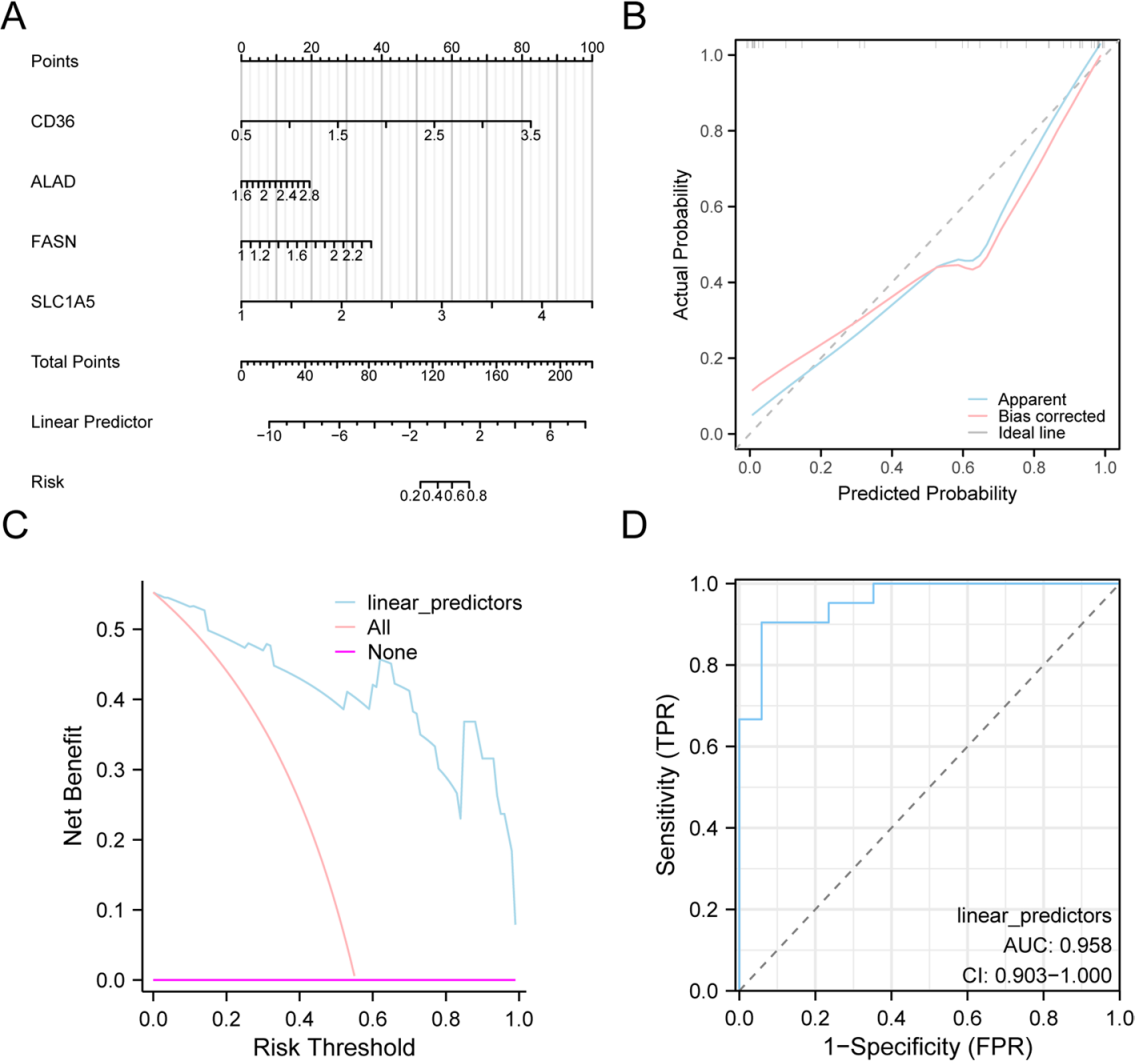
Construction and validation of the therapeutic monitoring nomogram. **A** Nomogram for predicting treatment response based on four key genes. **B** Calibration curve for the nomogram. **C** DCA for the nomogram. **D** ROC curve analysis of the logistic regression model. *DCA* Decision Curve Analysis, *ROC* Receiver Operating Characteristic; AUC > 0.9 had high accuracy, *AUC* Area Under the Curve

To validate the therapeutic monitoring model, we first constructed a nomogram using the four key genes from the GSE225620 dataset to visualize their predictive contributions (Fig. 6A). The nomogram revealed that *SLC1A5* expression had the most substantial impact on the model’s predictive score compared to the other variables.

Next, we assessed the model’s accuracy and discriminatory power using a calibration curve (Fig. 6B). This analysis compares the model’s predicted probabilities against the actual observed outcomes. The resulting curve showed only a minor deviation from the ideal diagonal line, indicating a high degree of calibration and reliable performance.

Furthermore, DCA was performed to evaluate the clinical utility of the key gene-based model (Fig. 6C). The DCA curve demonstrated that across a wide range of threshold probabilities, the model provided a greater net benefit than the strategies of treating all or no patients. This result underscores the model’s potential for clinical decision-making.

Finally, we evaluated the model’s ability to distinguish between pre- and post-treatment samples by plotting a ROC curve for the linear predictors derived from the logistic regression model (Fig. 6D). The resulting curve confirmed the model’s robust discriminatory performance in the GSE225620 dataset.

### Characterization and interrelation of key genes

**Fig. 7:**
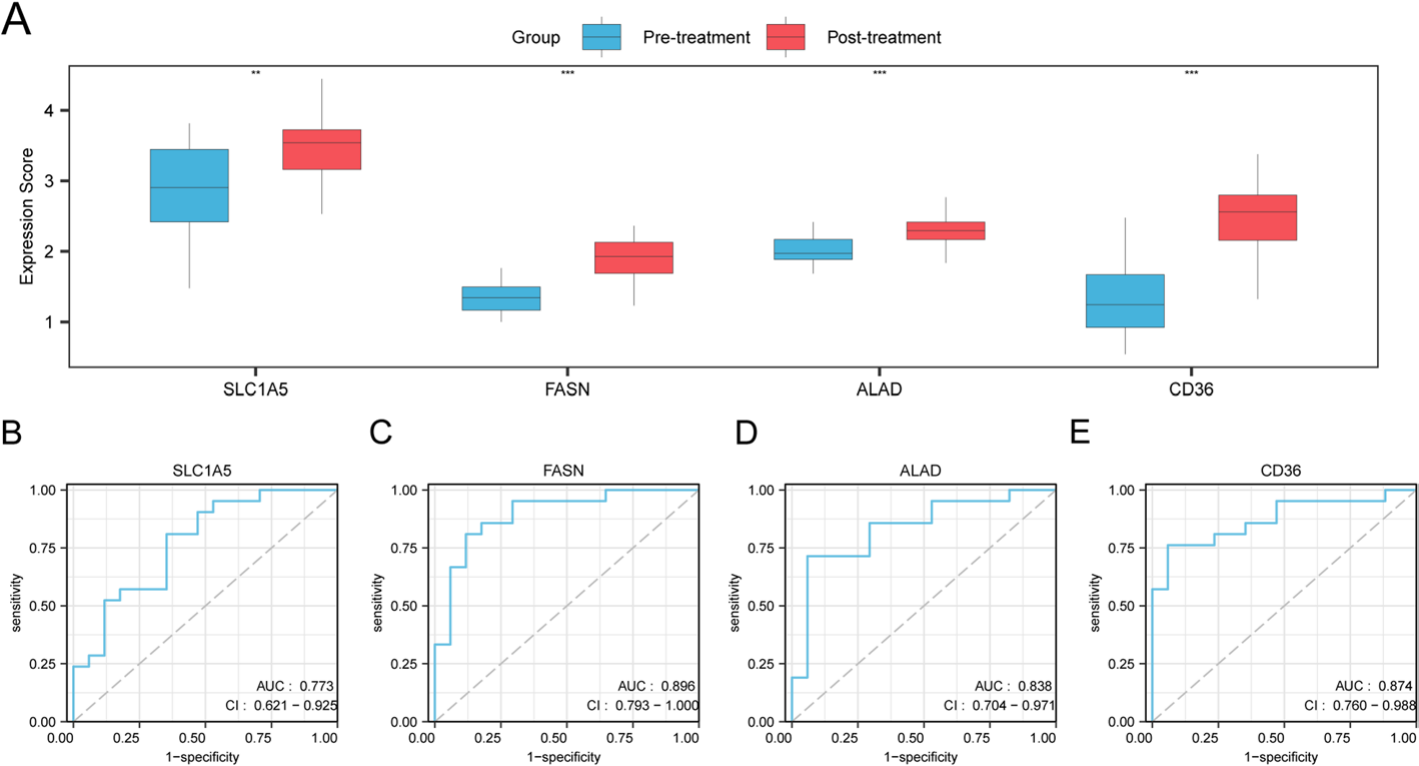
Expression and diagnostic value of the four key genes. **A** Box plots showing the differential expression of *SLC1A5*,* FASN*,* ALAD*,* and CD36* between Pre-treatment and Post-treatment groups. **B**–**E** ROC curves evaluating the predictive performance of *SLC1A5* (**B**), *FASN* (**C**), *ALAD* (**D**), and *CD36* (**E**). (*** *p* < 0.001, ** *p* < 0.01)

First, to evaluate the key genes as potential treatment-response biomarkers, we assessed their differential expression between pre- and post-treatment groups in the GSE225620 dataset. This comparison revealed that all four genes were expressed at significantly different levels (Fig. 7A). Specifically, *SLC1A5* showed significant differential expression (*P* < 0.01), while FASN, ALAD, and *CD36* exhibited a highly significant difference (*P* < 0.001). We then evaluated the capacity of each gene to distinguish between the two treatment states by performing Receiver Operating Characteristic (ROC) analysis. The Area Under the Curve (AUC) for all four genes ranged between 0.7 and 0.9, indicating strong discriminatory power (Fig. 7B–E).

**Fig. 8:**
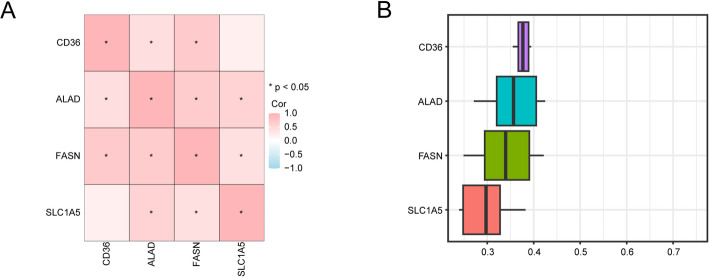
Correlation and functional similarity analysis of key genes. **A** Correlation analysis between Key Genes. **B** Functional similarity analysis of Key Genes. The absolute value of correlation coefficient (r value) below 0.3 was weak or no correlation, 0.3–0.5 was weak correlation, 0.5–0.8 was moderate correlation, and above 0.8 was strong correlation

Next, to investigate the co-expression patterns among these genes, we conducted a correlation analysis based on their expression profiles. The resulting heatmap revealed positive correlations between several key genes, with the most notable associations observed between *FASN* and *CD36*, and between *SLC1A5* and *ALAD* (Fig. 8A).

Finally, to explore their functional relationships, we quantified the semantic similarity of the four key genes based on their GO terms using the GOSemSim R package. This analysis, visualized in box plots, showed that *CD36* possessed the highest degree of functional similarity when compared with the other key genes (Fig. 8B).

### Immune infiltration analysis

To characterize the tumor immune microenvironment, we quantified the infiltration abundance of 28 immune cell types in the GSE225620 dataset using the single-sample Gene Set Enrichment Analysis (ssGSEA) algorithm.

**Fig. 9:**
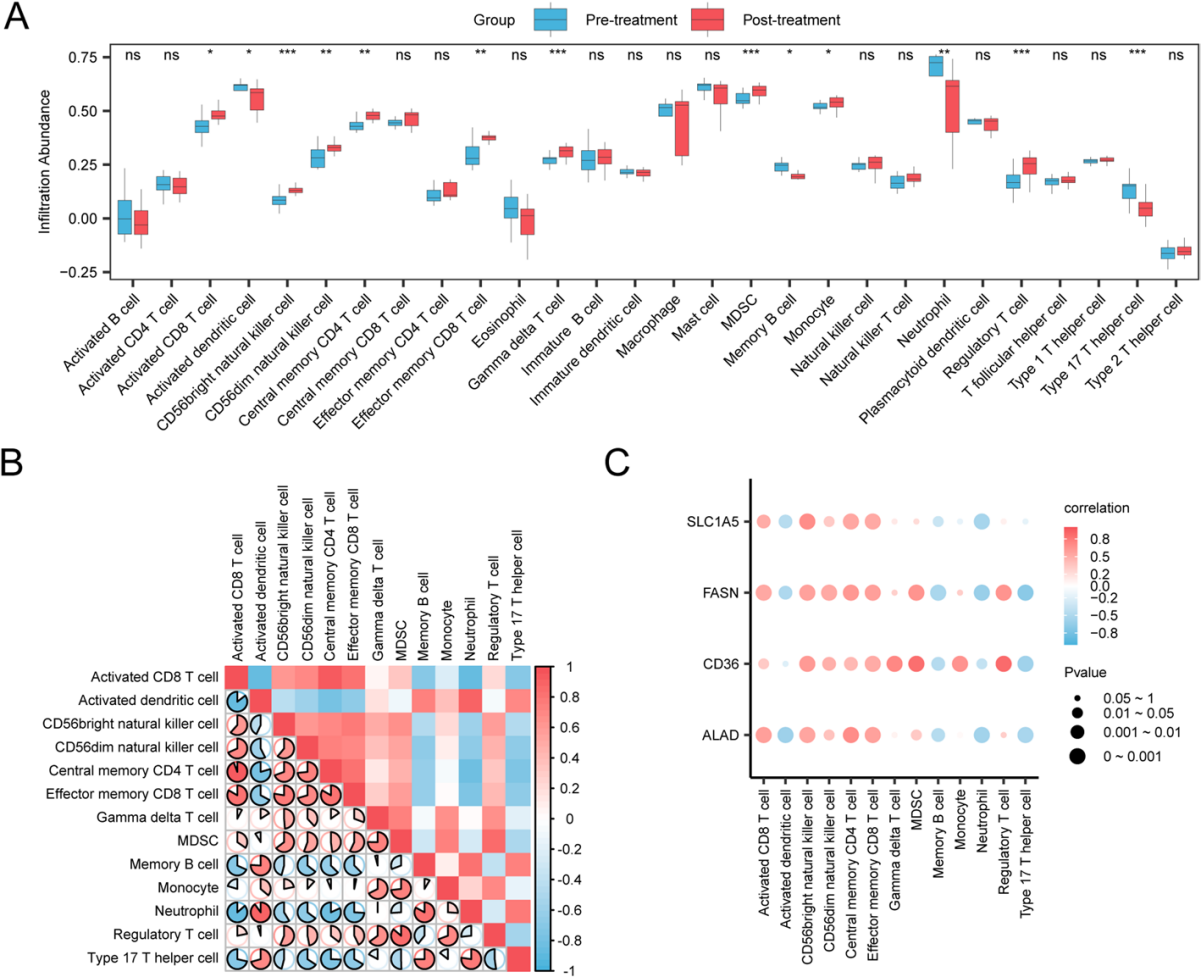
Immune Infiltration Analysis by ssGSEA Algorithm. **A** Comparison of the infiltration abundance of 28 immune cell types between Pre-treatment and Post-treatment groups. **B** Correlation heatmap of 13 differentially infiltrated immune cell types. **C** Bubble plot showing the correlation between the four key genes and immune cell infiltration. In panel A, asterisks indicate the level of statistical significance (ns *p* ≥ 0.05, * *p* < 0.05, ** p < 0.01, *** *p* < 0.001). In panel C, the size of the bubble represents the p-value. ssGSEA, single-sample Gene-Set Enrichment Analysis

First, we compared immune cell infiltration levels between the pre- and post-treatment groups. This analysis revealed statistically significant differences (*P* < 0.05) in the abundance of 13 immune cell types, including key populations such as Activated CD8+ T cells, Central memory CD4+ T cells, Regulatory T cells, and Myeloid-derived suppressor cells (MDSCs) (Fig. 9A).

Next, a correlation heatmap was generated to visualize the relationships among these 13 differentially infiltrated immune cell types (Fig. 9B). The results showed strong positive correlations among most cell populations, with the most robust association observed between Activated CD8+ T cells and Central memory CD4+ T cells (*r* = 0.943).

Finally, we investigated the relationship between our four key genes and immune cell infiltration by generating a correlation bubble plot (Fig. 9C). This analysis revealed numerous positive correlations, the strongest of which was between the expression of *CD36* and the infiltration of Regulatory T cells (*r* = 0.875).

**Table 1:** GEO microarray chip information

	GSE225620
Species	Homo sapiens
Samples in post-treatment group	21
Samples in pre-treatment group	17
PMID	PMID:38,951,894

*GEO* Gene Expression Omnibus; Post-treatment, Non-Small-Cell Lung Cancer

**Table 2:** Results of GO and KEGG enrichment analysis for BAM&IRDEGs

Ontology	ID	Description	GeneRatio	BgRatio	p value	*p*.adjust
BP	GO:0006631	Fatty acid metabolic process	24/109	395/18,800	4.41315E-18	1.54372E-14
BP	GO:0046395	Carboxylic acid catabolic process	18/109	238/18,800	2.19593E-15	3.2193E-12
BP	GO:0016054	Organic acid catabolic process	18/109	242/18,800	2.94241E-15	3.2193E-12
BP	GO:0044282	Small molecule catabolic process	21/109	376/18,800	3.6813E-15	3.2193E-12
BP	GO:0044242	Cellular lipid catabolic process	17/109	219/18,800	9.09363E-15	6.36191E-12
CC	GO:0005759	Mitochondrial matrix	22/109	473/19,594	1.42121E-14	3.36827E-12
CC	GO:0005777	Peroxisome	11/109	141/19,594	3.79911E-10	3.00129E-08
CC	GO:0042579	Microbody	11/109	141/19,594	3.79911E-10	3.00129E-08
CC	GO:0005778	Peroxisomal membrane	7/109	64/19,594	6.49354E-08	3.07794E-06
CC	GO:0031903	Microbody membrane	7/109	64/19,594	6.49354E-08	3.07794E-06
MF	GO:0016620	Oxidoreductase activity, acting on the aldehyde or oxo group of donors, NAD or NADP as acceptor	6/109	38/18,410	8.87234E-08	3.97481E-05
MF	GO:0016903	Oxidoreductase activity, acting on the aldehyde or oxo group of donors	6/109	46/18,410	2.89694E-07	6.48915E-05
MF	GO:0033293	Monocarboxylic acid binding	7/109	81/18,410	5.092E-07	7.60405E-05
MF	GO:0033218	Amide binding	13/109	402/18,410	7.42111E-07	8.31164E-05
MF	GO:0016614	Oxidoreductase activity, acting on CH-OH group of donors	8/109	140/18,410	1.82135E-06	0.000163193
KEGG	hsa01212	FATTY acid metabolism	10/96	57/8538	5.59751E-10	1.49454E-07
KEGG	hsa00620	Pyruvate metabolism	9/96	47/8538	1.88072E-09	2.51076E-07
KEGG	hsa05161	Hepatitis B	14/96	163/8538	3.01452E-09	2.68293E-07
KEGG	hsa05417	Lipid and atherosclerosis	15/96	216/8538	1.46388E-08	9.77142E-07
KEGG	hsa00020	Citrate cycle (TCA cycle)	7/96	30/8538	3.00215E-08	1.60315E-06

*GO* Gene Ontology, *BP* Biological Process, CC Cellular Component, *MF* Molecular Function, *KEGG* Kyoto Encyclopedia of Genes and Genomes, *BAM&IRDEGs* Bile Acid Metabolism&Invasion-Related Differentially Expressed Genes

## Discussion

NSCLC is the leading cause of cancer-related mortality. Despite advancements in treatment, the 5-year survival rate remains suboptimal, underscoring the urgent need to elucidate the underlying molecular mechanisms and identify novel therapeutic targets. This study employed a comprehensive bioinformatics approach to investigate the intricate interplay among bile acid metabolism (BAM), invasion-related genes, and the tumor immune microenvironment in NSCLC. Our investigation revealed that among 4615 differentially expressed genes (DEGs)—comprising 2679 upregulated and 1936 downregulated genes—109 were associated with both bile acid metabolism and invasion (designated as BAM&IRDEGs). These genes may represent critical regulators of NSCLC development and progression.

The identification of these BAM&IRDEGs is of particular interest. While the role of bile acids in carcinogenesis is complex and context-dependent, emerging evidence implicates their involvement in various malignancies, including colorectal, liver, and pancreatic cancers [30–32]. Beyond their canonical function in lipid digestion, bile acids are now recognized as signaling molecules that modulate cell proliferation, apoptosis, inflammation, and angiogenesis—all hallmarks of tumorigenesis [33]. The therapeutic potential of these BAM&IRDEGs warrants further exploration. For instance, ASPH overexpression in hepatocellular carcinoma (HCC) correlates significantly with tumor aggressiveness and metastasis. Notably, MO-I-1100, a small-molecule inhibitor of ASPH, has been demonstrated to inhibit the proliferation, migration, invasion, and colony formation of HCC cells [34]. Similarly, *IDH2* is significantly overexpressed in triple-negative breast cancer (TNBC) and is associated with an unfavorable prognosis. Consistent with this, silencing *IDH2* expression has been shown to suppress TNBC cell proliferation and invasion [35]. Therefore, preclinical validation of these targets using in vitro and in vivo models is an essential next step.

Pathway enrichment analysis revealed that the identified BAM&IRDEGs are significantly enriched in pathways related to fatty acid metabolism and bile acid degradation. These findings suggest a crucial role for these metabolic pathways in the pathogenesis of NSCLC. The concept of targeting metabolic reprogramming in cancer has gained considerable traction. For example, the *FASN* inhibitor TVB-3664 has shown significant anti-cancer activity in colorectal cancer models, an effect correlated with the upregulation of *CD36*, suggesting a compensatory mechanism involving increased fatty acid uptake [36]. Our results, which demonstrate the enrichment of BAM&IRDEGs in lipid metabolism-related pathways, suggest that targeting key enzymes or metabolites within these pathways may represent a promising therapeutic avenue for NSCLC.

Our analysis also revealed significant differences in the infiltration abundance of 13 immune cell types within the NSCLC microenvironment. A strong positive correlation was observed between activated CD8+ T cells and central memory CD4+ T cells (*r* = 0.943). These findings underscore the critical role of the tumor immune microenvironment in NSCLC progression. The tumor microenvironment is a complex ecosystem where diverse immune cells interact to influence tumor growth and metastasis. For instance, activated CD8+ T cells exert potent anti-tumor effects [37], whereas regulatory T cells can suppress anti-tumor immunity [38].Elucidating the functional roles of different immune cell subsets will provide a more comprehensive understanding of tumor immune evasion mechanisms.

Through LASSO regression and Support Vector Machine (SVM) analyses, we identified 6 and 20 key genes, respectively, with an intersection of 4 genes, including *CD36* and *SLC1A5*. The therapeutic monitoring model constructed using these four genes demonstrated robust performance across both training and validation sets, highlighting their potential as biomarkers for therapeutic monitoring. Notably, the primary clinical utility of this model lies in predicting therapeutic efficacy, monitoring treatment response, and guiding individualized treatment decisions for NSCLC patients. Its application for initial diagnosis would necessitate further validation in studies designed to distinguish patients from healthy controls or to stratify disease stages.

Our findings suggest a critical interplay between tumor metabolism and immune infiltration, pointing toward several potential mechanistic axes. One is a metabolic-immune reprogramming axis, wherein lipid metabolism aberrations directly impact immune cell function [39]. For example, a lipid metabolism-inflammation pathway involving key genes such as *CD36* may regulate fatty acid utilization, thereby shaping the local inflammatory and immune landscape [40]. Another is a bile acid signaling-immune regulation axis. Bile acids can activate receptors like *TGR5*, which has been shown to drive NSCLC cell growth via the *JAK2/STAT3* pathway [14]. Given that *STAT3* is a critical regulator of immune responses in the TME, bile acid signaling in tumor cells could indirectly modulate immune cell infiltration.

This framework provides a model for how key genes such as *CD36* and *SLC1A5* may facilitate immune evasion. *CD36*, a scavenger receptor, plays a direct role in T-cell exhaustion. Studies have shown that *CD36* on the surface of CD8 + T cells promotes the uptake of oxidized lipids from the tumor microenvironment, which triggers a signaling cascade leading to T-cell dysfunction and an exhausted phenotype [40]. This offers a direct mechanism by which *CD36*-mediated lipid metabolism contributes to tumor immune escape.

*SLC1A5* encodes an amino acid transporter primarily responsible for glutamine uptake. A specific *SLC1A5* variant has been identified as a mitochondrial glutamine transporter critical for driving cancer metabolic reprogramming. By transporting glutamine directly into mitochondria, this variant enhances the metabolic fitness of tumor cells [41], likely creating a nutrient-deprived, immunosuppressive microenvironment that hinders T-cell function. This mechanism is consistent with the CD8+ T cell exclusion observed in our analysis [42, 43].

This study has several limitations that should be acknowledged. First, our findings are derived from bioinformatics analyses and require experimental validation through in vitro and in vivo studies. Second, the reliance on a single cohort with a limited sample size may affect the robustness of our conclusions. To enhance the generalizability of our findings, future research should incorporate multi-center clinical samples and expand the sample size. The challenge of small sample sizes in machine learning could also be addressed by employing advanced computational methods, such as Generative Adversarial Networks (GANs). As reviewed by Ai et al., GANs can augment data by generating realistic synthetic gene expression profiles, thereby improving the generalizability of predictive models [44]. Furthermore, the use of whole-blood transcriptomics, while non-invasive, may not fully capture the molecular events within the tumor microenvironment. Lastly, our analysis is potentially confounded by batch effects inherent in public datasets.

A promising future direction involves translating these findings into clinical applications, particularly through liquid biopsies. As recent updates in pediatric oncology highlight, liquid biopsies are powerful tools for diagnosis, treatment monitoring, and relapse detection [45]. If the expression of *SLC1A5* or *CD36* can be reliably measured in circulating components such as cfDNA or cfRNA, they could serve as part of a non-invasive panel for risk stratification and dynamic monitoring. Concurrently, based on the evidence linking fatty acid and glutamine metabolism to tumor progression, therapeutic strategies targeting *SLC1A5* or *CD36* could be integrated with existing chemotherapy or immunotherapy to potentially enhance therapeutic efficacy [46–48].

In conclusion, this study integrated multiple bioinformatics approaches to characterize the interplay between bile acid metabolism, invasion-related genes, and the immune microenvironment in NSCLC. We identified key genes, notably *SLC1A5* and *CD36*, that are closely associated with NSCLC progression and established a therapeutic monitoring model with excellent predictive capability. These results not only deepen our understanding of the molecular mechanisms underlying NSCLC but also provide a foundation for developing novel diagnostic and therapeutic strategies.

## Supplementary Information

Supplementary material 1.

## Data Availability

The dataset analyzed during the current study is available in the Gene Expression Omnibus (GEO) repository. The data can be accessed through GEO accession number GSE225620 at the following link: https://www.ncbi.nlm.nih.gov/geo/query/acc.cgi? acc=GSE225620. All other data generated or analyzed during this study are included in this published article and its supplementary information files.
